# Dr. Arthur H. Pellette

**Published:** 1912-08

**Authors:** 


Dr. Arthur H. Pellette of Whitney Point, N. Y., a graduate of the N. Y. Homeopathic College, 1880, died at Binghampton, July 6.

